# Application of a single-flicker online SSVEP BCI for spatial navigation

**DOI:** 10.1371/journal.pone.0178385

**Published:** 2017-05-31

**Authors:** Jingjing Chen, Dan Zhang, Andreas K. Engel, Qin Gong, Alexander Maye

**Affiliations:** 1 Department of Biomedical Engineering, School of Medicine, Tsinghua University, Beijing, China; 2 Department of Psychology, School of Social Sciences, Tsinghua University, Beijing, China; 3 Department of Neurophysiology and Pathophysiology, University Medical Center Hamburg-Eppendorf, Hamburg, Germany; National University of Defense Technology College of Mechatronic Engineering and Automation, CHINA

## Abstract

A promising approach for brain-computer interfaces (BCIs) employs the steady-state visual evoked potential (SSVEP) for extracting control information. Main advantages of these SSVEP BCIs are a simple and low-cost setup, little effort to adjust the system parameters to the user and comparatively high information transfer rates (ITR). However, traditional frequency-coded SSVEP BCIs require the user to gaze directly at the selected flicker stimulus, which is liable to cause fatigue or even photic epileptic seizures. The spatially coded SSVEP BCI we present in this article addresses this issue. It uses a single flicker stimulus that appears always in the extrafoveal field of view, yet it allows the user to control four control channels. We demonstrate the embedding of this novel SSVEP stimulation paradigm in the user interface of an online BCI for navigating a 2-dimensional computer game. Offline analysis of the training data reveals an average classification accuracy of 96.9±1.64%, corresponding to an information transfer rate of 30.1±1.8 bits/min. In online mode, the average classification accuracy reached 87.9±11.4%, which resulted in an ITR of 23.8±6.75 bits/min. We did not observe a strong relation between a subject’s offline and online performance. Analysis of the online performance over time shows that users can reliably control the new BCI paradigm with stable performance over at least 30 minutes of continuous operation.

## Introduction

One of the most widely used visual BCI paradigms, featuring high classification accuracy and information transfer rate (ITR), is based on steady-state visual evoked potentials (SSVEP). The SSVEP is an electrophysiological brain response to periodically changing properties of a visual stimulus [1]. The frequency response is narrow-banded and follows the stimulation frequency (and its harmonics) up to at least 90 Hz [2].

The majority of the SSVEP BCI studies has utilized a visual spatial attention paradigm in which the visual interface consists of a group of luminance- or contrast modulated flickering stimuli placed at different locations. Each BCI command is usually associated with a visual stimulus flickering at a distinct frequency. The users operate the system by shifting their attention overtly or covertly to one of these stimuli [3–6], leading to an enhanced SSVEP response at the corresponding frequency. To date, high-performance SSVEP-BCIs have been achieved mainly by employing an overt attention task [4] with flickering stimuli at a relatively low frequency (i.e. < 20 Hz) which maximizes both the SSVEP responses and the attention-related enhancement. However, directly gazing at these low-frequency flickering stimuli (overt attention) can easily cause fatigue and even runs the risk of eliciting photic epileptic seizures [7–10]. This constitutes a severe limitation of the long-term application of SSVEP-BCIs. To alleviate visual strain, substantial efforts have been devoted towards the development of covert attention-based BCIs [5,10–12], high-frequency SSVEP BCIs [13,14,7,10,15], and hybrid SSVEP BCIs integrating other visual BCIs paradigms [16,17]. Nevertheless, the reported systems were either associated with a limited number of BCI commands or showed relatively low information transfer rates.

A recently proposed SSVEP BCI paradigm, in which control channels are coded by their spatial position rather than their flicker frequency or phase [18], may provide an alternative route towards a practical SSVEP BCI with reduced visual strain. Instead of assigning each BCI command a distinct flicker stimulus, this BCI paradigm employs only a single flicker, and BCI commands are associated with non-flickering targets surrounding the SSVEP-generating flicker stimulus. This approach rests on the observation that different relative positions between the flicker stimulus and the foci of overt attention result in distinct topographies of the SSVEP responses. The first offline study on 12 healthy volunteers revealed that up to 9 targets can be recognized with >90% accuracy (4 seconds trial duration). Besides the promising performance, this spatially coded SSVEP BCI opens new possibilities for application design. As the attention targets were non-flickering and did not require any other specific designs (e.g. color, contrast, etc.), the flickering stimulus can be integrated in a variety of different background contexts, e.g. naturalistic images, gaming environments, etc.

To demonstrate the integration of this spatially coded SSVEP BCI in a user interface, and to put the paradigm to the test of an online BCI, we developed an application scenario in which users had to navigate a two-dimensional landscape of a simple computer game. By looking at the 4 sides of a flickering square at the screen center, they could shift a virtual landscape and thereby reach a given goal location. All users but one achieved reliable control of the movements in the virtual environment and reached the goal location within the given time limit. We evaluate their performance during the training session, in the online session, the relation between the two performance measures, and the stability of the online performance over 30 minutes of operating the BCI. Our study demonstrates that this new SSVEP BCI type operating in closed-loop mode enables efficient long-term communication with the environment.

## Methods

### Subjects

Twelve healthy subjects (7 females and 5 males, mean age: 23.5 years, age range: 19–32) participated in this study. All of them had normal or corrected-to-normal vision and were free of neurological and ophthalmological disorders. One subject reported a mild conjunctivitis, but the resulting increase in eye blinks remained in the range of the other subjects. In accordance with the Declaration of Helsinki, all subjects provided written informed consent prior to participating in the experiments and received financial compensation for their participation. This study was approved by the ethics committee of the medical association of the city of Hamburg, Germany.

Each subject completed a training session that was immediately followed by an online session. An experiment lasted about 1.5 hours.

### Visual stimulation

**Fig 1:**
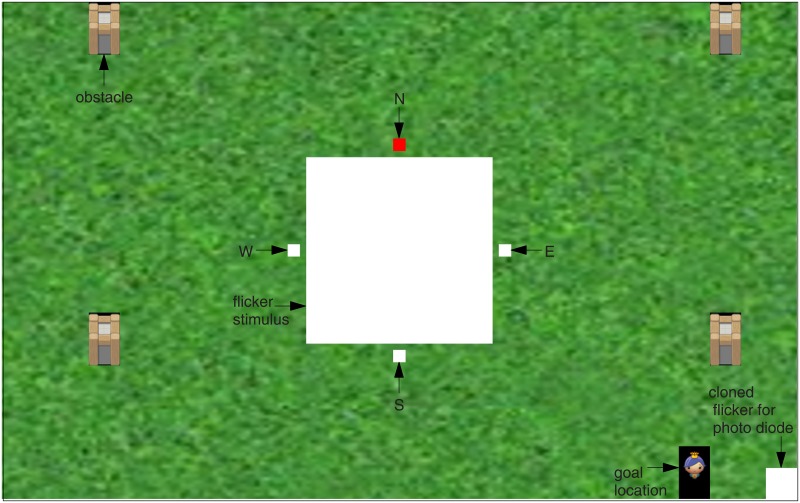
Annotated screenshot of the visual stimulation. The small white squares labeled N, W, S and E indicate where the subjects should focus their gaze in order to move the landscape in the respective direction. In the training session, the target was cued by turning the color to red (in this example, subjects should look at the N target).

The visual input presented to the subjects consisted of the BCI stimulus on top of a 2-dimensional map that displayed the game environment (see Fig 1).

The BCI stimulus in the center of the screen was composed of a flickering white square (12x12 cm, 13.7° visual angle) surrounded by 4 small, static (i.e., non-flickering) squares (0.7x0.7 cm, 1.6° visual angle) outside the four edges of the central square (north-N, east-E, west-W, south-S). The flickering square (*f*_*stim*_ = 15Hz) elicited the SSVEP, and the four small squares indicated the target locations where the users should direct their gaze to. In the training session, the target indicators were white, and the gaze direction of the subjects was cued by coloring the respective target red. During the online session, all four target indicators were red. The BCI stimulus resembles the one we used in our previous offline study [18] when used with 4 classes. Main differences are the circular shape and the doubled size (~26 cm diameter, 27° visual angle) that was used there as well as the location of the gaze targets at the fringe but inside the flickering circle. Although a parametric exploration of the effect of the stimulus size and shape (and other parameters) on the classification accuracy is needed, we suggest that a follow-up offline study is more appropriate for this purpose.

The display of the game environment covered the remaining screen area and consisted of a patterned green background with obstacles that were visualized by icons of towers and the goal position, represented by the icon of a princess. The environment remained static during the training session. In the online session however, a shift of the environment in response to the user’s command was animated with 2 s duration.

The stimulus was presented on an LCD computer monitor (24 in, HP EliteDisplay E241i, 1280x800 pixel resolution, 50 cm viewing distance).

### Experimental paradigm

In the training session, subjects were cued to fixate all 4 target locations in random order. Each target was cued for 4 seconds, and EEG was recorded simultaneously. After looking at all 4 targets, the stimulus disappeared for 1.5 s, before the next random sequence of targets was presented. Subjects could blink or swallow in this interval. Fifty sequences were presented in a block. Then a short break of 3–5 min was administered. Two training blocks were recorded in total. A training session lasted approximately 35–40 minutes. Compliance with the task was checked intermittently using a webcam mounted at the top of the screen that was directed at the subject’s face.

In the online session, the user was requested to move the game landscape by gazing at the respective target indicators (N, E, W, S) so that the goal position (visualized by the cartoon princess) moved step by step towards the center of the screen. After a successful recognition of a movement direction that the user intended, the whole landscape moved one step in the respective direction. During the animated movement of the environment, subjects could plan the next step and blink or swallow. In each round of the game, the initial goal position was always 12 steps away from the center; 6 steps in x-direction and 6 steps in y-direction. This means that goal positions were always in one of the corners of the environment, and that at least 12 steps were needed to reach it. In order to handle users who could not reliably control the BCI, we introduced an upper step limit. If the subjects could not reach the target in 25 steps, they lost the game, and a new round was started for the next trial. If the subjects reached the target in less than 25 steps, an encouraging message was shown, and the game continued with the next trial. Initial goal positions were randomized across trials.

The stimulation software was written in MATLAB (The Mathworks, Natick, MA, USA) using the Psychophysics Toolbox extension [19–21]. Software for stimulation, EEG recording and the BCI ran on a regular desktop PC (HP Compaq Elite 8300).

### EEG recording and data processing

A 32-channel EEG was recorded continuously using BioSemi’s ActiveTwo AD-box (BioSemi Instrumentation, Amsterdam, The Netherlands). Electrodes were placed according to the 10–20 international electrode position system. The lab streaming layer (LSL, https://github.com/sccn/labstreaminglayer) was used to record EEG data and to synchronize them with the experimental condition. Accurate synchronization of the EEG data with the flicker stimulus on the screen was achieved by a photodiode in the lower right corner of the screen that was driven by a smaller clone of the central flickering square and that was connected to the trigger input of the EEG amplifier. All experiments were conducted in a typical office room without any electromagnetic shielding.

EEG data from the training session were preprocessed offline by a 1–80 Hz bandpass filter. Eye blink and muscle artifacts were removed semi-automatically. In particular, episodes with EOG and muscle artifacts were detected automatically, verified by visual inspection and rejected manually. The first 500 ms after stimulation onset were discarded, resulting in an effective trial length of 3.5 s. The data were then downsampled from 1024 Hz to 512 Hz. The two training datasets per subject were concatenated and used to calculate the offline classification accuracy as well as to train the classifier.

In the online session, data epochs corresponding to the stimulation intervals were extracted based on the event triggers that had been generated by the stimulus program. Then a 1–80 Hz bandpass filter was applied to the signal. The CCA features were calculated as described below and forwarded to the classifier which then recognized the movement direction that the user had intended. This output was used to shift the landscape in the respective direction.

### Feature extraction and classification

As the stimulation setup was designed to elicit distinct SSVEP power distributions on the subject’s scalp for each target direction, a straightforward method for inferring the attended target is to apply spatial filters on the EEG data that maximize the output for the respective target, and to use these filter outputs as feature vectors for classification. Details of the method are given in [18], but the main processing steps will be described here.

We used canonical correlation analysis (CCA, [22]) to determine spatial filters *A*_*c*_ and *B*_*c*_ that maximize the canonical correlation *r*_*c*_ = [*ρ*_1_ … *ρ*_*M*_] of the filtered EEG data *A*_*c*_*X* with a filtered reference signal *B*_*c*_*Y* for each of the four target directions *c* = 1 … 4. To obtain filters *A*_*c*_ and *B*_*c*_, we concatenated all trials from the training session when the subject was cued to look at target location *c* and calculated the CCA with the *M* = 6 dimensional reference signal

$$Y=\left[\begin{array}{c} \text{sin}\left(2\pi f_{stim}t\right)\\ \text{cos}\left(2\pi f_{stim}t\right)\\ \text{sin}\left(4\pi f_{stim}t\right)\\ \text{cos}\left(4\pi f_{stim}t\right)\\ \text{sin}\left(6\pi f_{stim}t\right)\\ \text{cos}\left(6\pi f_{stim}t\right) \end{array}\right].$$

Then, for each trial in the training data, a feature vector was composed by calculating the canonical correlations *r*_*c*_ (using the previously determined filters *A*_*c*_ and *B*_*c*_) for all four targets and concatenating them: [*r*_1_ *r*_2_ *r*_3_ *r*_4_]. These feature vectors together with the corresponding target locations were used to train a multi-class linear discriminant analysis classifier [23].

In the online session, feature vectors were calculated like for the training data, and the output of the classifier determined the direction in which the landscape was shifted.

### Calculating ITR

For the data from the training session, ITR was calculated using the equation from [7]:

$$ITR=\frac{60}{T}\left({\text{log}}_{2}\;C+P{\text{ log}}_{2}\;P+\left(1-P\right){\text{ log}}_{2}\frac{1-P}{C-1}\right)$$ (1)

Here, *P* is the classification accuracy, *C* the number of classes (*C* = 4) and *T* the effective trial length (*T* = 3.5s).

The user intention was not tracked during the online session; hence, the classification accuracy *P* is not known. We determined however a lower bound of the online ITR by using Eq 1 and estimating the online classification accuracy in the following way. Under the assumption that subjects followed the optimal strategy for approaching the target location, the classification accuracy can be derived from the success of each individual classification with respect to the goal. In each trial we therefore counted the number of steps that reduced the Euclidean distance to the goal position and divided it by the total number of steps made in this trial. This approach considers the performance in the same way no matter whether the goal position was finally reached or not. In fact we observed that several games were ‘lost’ when subjects were only one or a few steps away from the goal position, and the round was terminated because the fixed but arbitrary limit for the number of steps was reached. As subjects may not have followed the optimal strategy all the time, the true classification accuracy in the online session may have been higher than this estimate; therefore, the calculated online ITRs should be considered as a lower bound.

## Results

**Fig 2:**
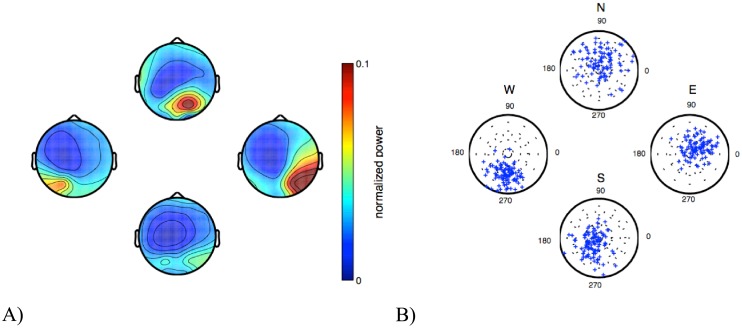
SSVEP responses for a typical subject. (A) SSVEP power topographies for the four target directions. (B) Phase of the SSVEP at electrode O1.

First we analyzed the SSVEP response when subjects fixated the four different target indicators during the training session. Fig 2 shows the corresponding SSVEP power topographies (A) and phase-amplitude plots (B).

The SSVEP power topographies (Fig 2A) show the typical power maximum over occipital and parietal areas. A systematic shift of the power maximum between right and left occipital areas for targets E and W can be observed. When subjects gazed at the east direction, the flicker stimulus appeared in the left visual hemifield; hence, an increase in the activity over the contralateral, i.e. right hemisphere, can be observed. The opposite relation holds for the west direction. When the flicker stimulus moves from a position above the horizontal midline to below, i.e. when the subjects shift the gaze from target S to N, the region of maximal SSVEP power shifts from an occipital position towards a centro-occipital region.

Polar plots of the single-trial SSVEP response at occipital electrode O1 can be seen in Fig 2B. Together, the plots show that distinct patterns emerge when subjects gaze at the different targets, suggesting that a BCI based on the classification of spatial SSVEP amplitude and phase responses is feasible.

Details of the classification accuracies and ITRs in the training phase and the online session are given in Table 1. The average classification accuracies across subjects are 96.9±1.64% and 87.9±11.4% respectively, corresponding to average ITRs of 30.1±1.8 bits/min and 23.8±6.75 bits/min respectively.

**Table 1:** Classification accuracies and ITRs for all subjects.

subject number	offline accuracy [%]	offline ITR [bits/min]	online accuracy [%]	online ITR [bits/min]
**1**	96.5	29.6	92.7	27.8
**2**	98.1	31.5	96.7	29.9
**3**	98.8	32.4	95.4	28.8
**4**	98.9	32.5	86.6	21.3
**5**	95.8	28.9	92.8	26.3
**6**	97.4	30.6	94.3	27.4
**7**	98.1	31.5	96.9	30.1
**8**	97.6	30.8	89.4	23.7
**9**	95.7	28.7	91.8	25.7
**10**	96.3	29.4	75.1	16.5
**11**	96.4	29.5	57.1	6.62
**12**	93.1	26.2	85.7	21.6
**mean**	96.9	30.1	87.9	23.8
**standard deviation**	1.64	1.80	11.4	6.75

To further evaluate the performance during the online session, we determined for each subject the number of games won, the average number of steps they needed to reach the goal position and the time spent for all games (cf. Table 2). Out of 16 game rounds in total, subjects won on average 12.7, taking 16.4 steps per game and spending 33 minutes for all rounds together (won and lost). Whereas most subjects won 15 or all 16 games, subjects 10 and 12 reached the goal position only in 10 and 13 games respectively, and subject 11 failed to achieve reliable control of the BCI.

**Table 2:** Quantitative evaluation of the online session. Session time is excluding breaks between trials.

subject number	number of successful games	mean step number to win a game	online session time [min]
**1**	16	13.8	28
**2**	16	13.6	28
**3**	16	14.3	29
**4**	15	17.9	36
**5**	15	15.8	32
**6**	16	14.4	30
**7**	16	13.3	27
**8**	15	17.3	33
**9**	15	15.5	31
**10**	10	19.9	40
**11**	2	24.4	49
**12**	13	17	34
**mean**	12.7	16.4	33
**standard deviation**	4.09	3.21	6.26

**Fig 3:**
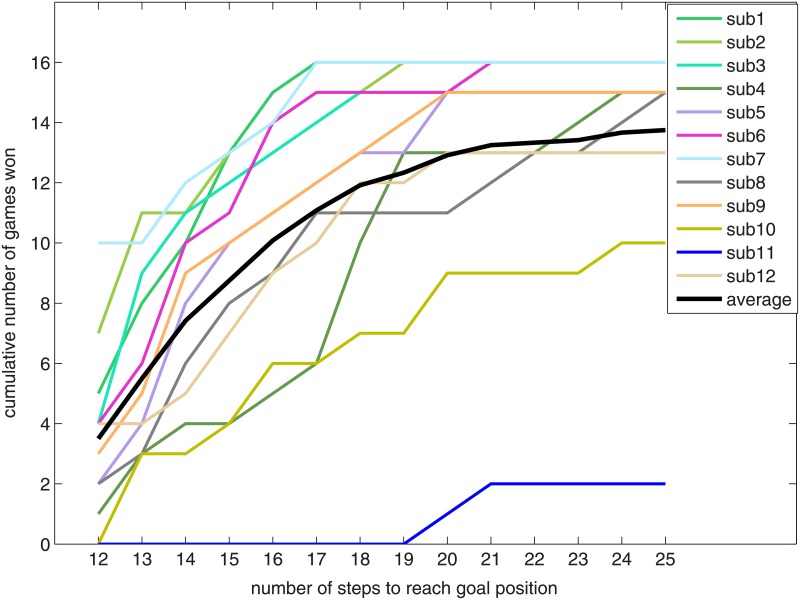
Number of steps that each participant needed to reach the goal position.

We also analyzed the distribution of the number of steps that subjects needed to finish the game. Fig 3 visualizes the frequency at which each subject finished a game in a particular number of steps.

The majority of subjects could win the game with no more than 21 steps, indicated by the shallow increase of the average number of games won for more than 21 steps.

To assess whether the offline classification accuracy can predict the subject’s performance when operating the online system, we computed the correlation coefficient between offline and online classification accuracy. As the correlation is relatively low (r = 0.28), and the p-value does not reach a significance threshold (p = 0.39), the offline classification accuracy seems to have a low predictive value for the online performance. Even omitting the two potential outliers with the lowest online and offline accuracies respectively did not qualitatively change this observation.

**Fig 4:**
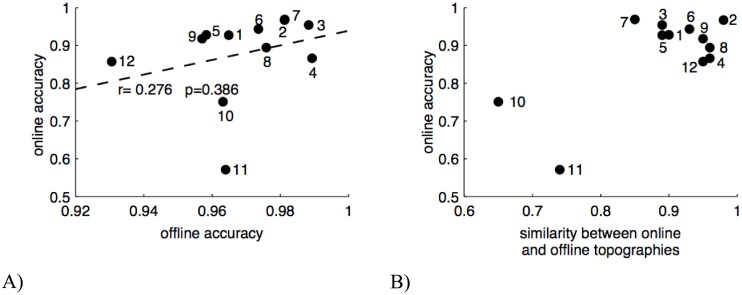
A) Correlation between the offline and online classification accuracies. B) Relation between online accuracy and the similarity of EEG topographies between offline and online session. Dots are labeled with the subject number.

From Table 1 it becomes evident that the performance of two subjects (10 and 11) in the online session is markedly lower than that of the other subjects. The performance of these two subjects in the offline session however is well within the range of the other subjects. In order to figure out possible causes for this performance drop, we calculated the grand average across all trials, irrespective of the target, for the offline session and for the online session separately and compared the two SSVEP topographies. We quantified the similarity between online and offline topographies by the Pearson correlation coefficient and plotted it together with the online classification accuracy in Fig 4B. Tellingly, this shows two clusters: one with the two subjects that have low online classification accuracy and low similarity between the topographies, and another one with the remaining subjects that have high online classification accuracy and high similarity between the topographies.

**Fig 5:**
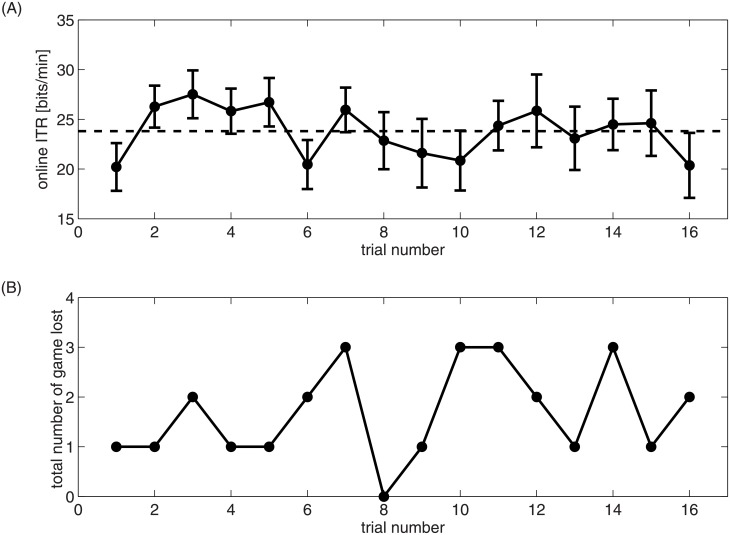
Online performance over time. Average ITR across subjects (A) and number of lost games (B) for each trial in the online session. Error bars show the standard error of the mean.

To reveal if and how fatigue affects the performance of the BCI system, we analyzed the development over time of the online classification accuracy (Fig 5A). Subjects started with a relatively low ITR in the first game round but quickly improved until the 7^th^ round (with a minor drop in the 6^th^ round). From then on, performance generally seems to become more variable but to stay at the same level. We used the number of lost games as an additional indicator for the efficiency of the BCI. We could not observe a trend over the trials of the online session though (see Fig 5B). In particular, we did not find an increase in the frequency of lost games towards the end of the session, which would be expected if fatigue had affected classification accuracy.

## Discussion

Our study demonstrates the application of a spatially coded SSVEP BCI in closed-loop mode for solving a 2-dimensional navigation task. A single flicker stimulus was utilized to elicit an SSVEP response, and the user’s gaze direction relative to the stimulus caused distinguishable power topographies that were automatically classified and used to control four movement directions. Analysis of the online performance shows that most users can efficiently solve the task, and that performance remains stable over more than 30 minutes of continuous operation.

A comparison between the estimate of the ITR from the training data and the ITR of the online system shows that the estimate is consistently higher, but that both values are nevertheless in the same range. We tested therefore whether effects of fatigue like decreased SSVEP power or increased power in the alpha frequency range during the online session might have caused this finding, but we could not observe a consistent change of these parameters between the training and online sessions (data not shown). Therefore, the ITR difference may have other causes. For example, subjects may have been more agitated in online mode, because it was more interesting to play the game, and success or failure to reach the goal position affected them emotionally. This may have impacted the SSVEP power and/or its spatial distribution, leading to lower classification accuracy than in the training session. We therefore quantified the similarity between the SSVEP topographies in the training and online session and observed a clear separation between the subjects that showed low classification accuracy in the online session and relatively low similarity between the topographies, and those subjects with high online classification accuracy and high similarity of the topographies. As the spatial distribution of the SSVEP response is the basis for the target classification, different distributions in the training and application phase may indeed causally affect the performance. In general, subjects may also have voluntarily or involuntarily deviated from the optimal trajectory which was stipulated for calculating ITRs in online mode. Therefore, it is reasonable to assume that the true online ITRs are in general more similar to the offline ITRs than the conservative approximations we made here.

Furthermore, we investigated whether the classification accuracy calculated offline on the training data is predictive for the performance of the system in online mode. Interestingly, we did not observe a strong correlation. Whereas all subjects achieved high offline classification accuracies (all well above 90%), there was substantial variation of their online performances. One explanation for the lacking correlation may be that the overall high offline accuracies lead to a ceiling effect that does not allow for comparisons with accuracies along the full range down to chance level. However, the current study corroborates the results of the offline analyses in our previous study [18].

To date, spatial navigation applications have mostly employed either motor imagery or SSVEP BCI paradigms. While motor imagery BCIs and their hybrid forms have achieved considerable progress [24–26], ITRs are comparatively low (approximately 6.8 bits/min in [25] and 11.9 bits/min in [26] using Eq 1). Although considerations about theoretical channel capacities up to 50 bits/min have been made [27], temporal averaging to consolidate classification results, the relative sluggishness of switching between mental states compared to the briskness of gaze shifts in SSVEP BCIs as well as the task demands keep the practical ITRs well below this mark. Besides, motor imagery BCIs heavily depend on the subject’s ability to enter and maintain distinct mental states and therefore are more susceptible to problems with ‘BCI illiteracy’ [28].

SSVEP-based BCIs provide an alternative solution for people who perform poorly with motor imagery BCIs. Whereas the spatially coded BCI we present here uses only a single flicker stimulus, conventional frequency-coded BCIs for 2D navigation typically employ 4 flicker stimuli with different frequencies to encode the 4 movement directions. For example, the systems described in [29–32] implemented the stimulus by 4 LEDs around the edges of a screen or on a panel that represent the commands to navigate a robot or a wheelchair. Only few of the relevant articles calculated the online ITR of the investigated BCI system, but from the data given in these articles, we estimated 9.9 bits/min in [29], 17.24 bits/min in [30], and ~20 bits/min in [31]. The ITR of 23.8 bits/min of the system we present here is well in this range. To our knowledge, the currently fastest system of this type achieved around 44.3 bits/min [32]. Whereas subjects typically reported no or only little fatigue after the experiment, their experience and the performance were assessed only in a few trials (2–5 trials [31,32]) or even in a single trial [29,30]. In contrast, testing performance by letting subjects repeatedly solve the same task over about 30 minutes allows us to arrive at a more reliable assessment of the long-term stability of the system. In our study, the overall performance stayed relatively stable with increasing trial-to-trial variability over time.

A common approach to reduce fatigue and other problems caused by directly looking into the flicker stimuli in frequency-coded SSVEP BCIs is to employ higher flicker frequencies. Coding the control channels by frequencies in the range from 34 to 40 Hz has proven to be successful in achieving reliable target classification while at the same time reducing visual strain [30–32]. It seems straightforward to employ higher flicker frequencies for the spatially coded SSVEP BCI we present in this article as well, and we will dedicate a separate study to the experimental validation.

Using only a single flicker stimulus considerably simplifies the stimulation setup and the user interface of SSVEP BCIs. This allows users to control the BCI in an intuitive yet reliable way. The fact that in a spatially coded SSVEP BCI the flicker stimulus appears always in the peripheral field of view suggests that visual fatigue may be reduced in comparison to conventional frequency-coded SSVEP BCIs, but a direct comparison of both approaches would be needed to test this hypothesis.
